# Linking *Populus euphratica* Hydraulic Redistribution to Diversity Assembly in the Arid Desert Zone of Xinjiang, China

**DOI:** 10.1371/journal.pone.0109071

**Published:** 2014-10-02

**Authors:** Xiao-Dong Yang, Xue-Ni Zhang, Guang-Hui Lv, Arshad Ali

**Affiliations:** 1 College of Ecological and Environmental Sciences, East China Normal University, Shanghai, China; 2 Tiantong National Forest Ecosystem Observations and Research Station, Chinese National Ecosystem Observation and Research Network, Ningbo, China; 3 Institute of Resources and Environment Science, Xinjiang University, Urumqi, China; 4 Xinjiang Key Laboratory of Oasis Ecology, Ministry of Education, Urumqi, China; Lakehead University, Canada

## Abstract

The hydraulic redistribution (HR) of deep-rooted plants significantly improves the survival of shallow-rooted shrubs and herbs in arid deserts, which subsequently maintain species diversity. This study was conducted in the Ebinur desert located in the western margin of the Gurbantonggut Desert. Isotope tracing, community investigation and comparison analysis were employed to validate the HR of *Populus euphratica* and to explore its effects on species richness and abundance. The results showed that, *P. euphratica* has HR. Shrubs and herbs that grew under the *P. euphratica* canopy (under community: UC) showed better growth than the ones growing outside (Outside community: OC), exhibiting significantly higher species richness and abundance in UC than OC (*p*<0.05) along the plant growing season. Species richness and abundance were significantly logarithmically correlated with the *P. euphratica* crown area in UC (*R^2^* = 0.51 and 0.84, *p*<0.001). In conclusion, *P. euphratica* HR significantly ameliorates the water conditions of the shallow soil, which then influences the diversity assembly in arid desert communities.

## Introduction

The hydraulic redistribution (HR) is defined as the movement of water from the moist to dry soil portions through roots of deep-rooted plants [1]–[3]. Through this process, water moves upward or laterally among different soil layers, and subsequently changes the water spatial pattern of soil [3], [4]. Thus, HR improves the fine roots survival rate of deep-rooted plants and protects the shallow-rooted plants growth in extremely dry environments [4]–[6]. In addition HR plays an important role in positive feedback between plants and soil moisture circulation [1], [7]–[10], it also benefits the plant rhizosphere nutrient absorption, community stability, and biodiversity maintenance [3], [11]–[20].

*Populus euphratica* Oliv., a deciduous plant with high tolerance to drought and saline conditions, and is a dominant species for the Tugai Forest (desert riparian forest) in the Ebinur desert. Pervious research findings have revealed that the water gradient of soil layers determines the occurrence of HR in deep-rooted plants [3], [4], [11], such as when the surface soils are extremely dry and the deeper soils are rich in available water [3], [4], [21]. *P. euphratica* developed a deep vertical root system for absorbing water in Chinese arid deserts due to effect of high wind [22]–[24]. Moreover, the annual precipitation of this site is less than 100 mm and the surface soils are extremely dry, whereas the groundwater level is relatively high because of glacial melt waters [22]. Hence, *P. euphratica* should possess HR.

In our previous study [22] and the one conducted by Hao et al. [23] employed the Ryel model [25], that was used to simulate HR of *P. euphratica.* According to the research findings of the above mentioned studies that *P. euphratica* has HR which reached a maximum point at 2∶30 am for a given day, and the total amount of HR decreased with changes in the plant growing season [22], [23]. But, those results were only based on water characteristics (water content and water potential) changes along the various soil layers, which are only obtained from model predictions. The experimental support of *P. euphratica* possessing HR is lacking. Here we investigate whether oxygen isotope tracking can be used to test whether the plants possess HR [1]–[4], [11], and this could provide the experimental support to the studies based on model simulations. As oxygen isotope does not fractionate during water transport through xylem vessels, whereas the ^18^O/^16^O values varied across the different soil layers [1], [26]–[28]. For example, the δ^18^O content continues to increase from deep to shallow soils based on fractionation. Thus, we can predict that if *P. euphratica* possess HR, *P. euphratica* roots can lift water containing lower δ^18^O content from groundwater and deep soils to shallow soils through xylem vessels, which can result in soil having lower δ^18^O content under the *P. euphratica* canopy (under community: UC) rather than outside the *P. euphratica* canopy (outside community: OC).

Annual precipitation in Ebinur desert is very scarce, which is insufficient for the basic physiological activity of plants, while the deep soils and shallow groundwater contains abundant water for plant growth in this region [29]. However root density of most plants decreases with increasing soil depth [22], [30], [31], and only a small number of deep-rooted plants are able to penetrate to the deeper soils or the water table to absorb water which limits water utilization and subsequently, decreases the species richness and abundance in the arid desert [32], [33]. So, if *P. euphratica* HR lifts water from the deeper soils or groundwater aquifer to shallow soils through its roots, this process can increase the water content of surface soils and afford more water for shallow-rooted shrubs and herbs. In this case, it can induce the plants assembly under *P. euphratica* canopy and subsequently maintain the biodiversity in arid deserts. Therefore, we hypothesize that, the shallow-rooted shrubs and herbs grow better in UC than in OC and there is a higher species richness and abundance in UC than in OC, because *P. euphratica* HR significantly improves the soil water content. But, the correlation between *P. euphratica* HR, plant growth condition and biodiversity in arid desert areas is poorly understood.

The objective of this study was to test for *P. euphratica* HR and to explain the effect of HR on plants growth condition and species biodiversity maintenance in the Ebinur desert. We predict the following: (1) δ^18^O content of the shallow soils in UC are higher than in OC; (2) shrubs and herbs of UC have higher growth condition than those of OC; and (3) *P. euphratica* HR influence species abundance and richness in arid desert.

## Materials and Methods

### Study site and Ethics Statement

The experimental site is located in the Ebinur Lake Wetland Nature Reserve (ELWNR) in the western margin of the Gurbantonggut Desert in Xinjiang Uygur Autonomous Region of China (44° 30′–45° 09′ N, 82° 36′–83° 50′ E). This site belongs to a tuyere zone of Alashankou, the annual wind days (days with wind speed ≧17 m/s) are more than 164 d, and the annual fresh gale hours are approximately 241 hr. The annual sunshine hours reach approximately 2800 hr, and the annual precipitation is less than 100 mm, whereas the potential evaporation is more than 1600 mm. In addition, the temperature in this area ranges from 44 to −33°C, with an average temperature ranging from 6 to 8°C, and an average temperature of the growing season is roughly 25°C. Due to the extremely dry conditions and sparse rainfall, the climate is classified as typical temperate continental arid [22].

No specific permissions were required for the described field studies in ELWNR. The ELWNR is owned and managed by the local government and the location including the site used for our experiment are not privately owned or protected in any way and thus a specific permit for not for-profit research is not required. The field studies did not involve endangered or protected plant species in this area.

### Study plot and samples collection

5×5 km (25 km^2^) typical plots of *P. euphratica* were chosen as our experimental plots, where 48 *P. euphratica* individuals are distributed and the vegetal coverage is 4%. This site has a moderate slope and sandy soil. The distribution of groundwater level in our experimental site ranges from 1.5 to 1.8 m. Moreover, sand dunes are present 3 km north of the site. Based on the terrain of the experimental site, we randomly selected three *P. euphratica* individuals with no differences in growth conditions (DBH, tree height and crown area) among those trees as the experimental (UC) group. The selected plant individuals were at least 6 m apart from each other to prevent mutual water transfer process among them. For comparison, we randomly selected three plots located closely to the selected three *P. euphratica* individuals as the control (OC) group, where no *P. euphratica* were growing and no groundwater table difference with UC group.

To distinguish the difference in water transport from deeper soil layers to surface soil layers between the UC and OC groups, three points in each two types of plots were randomly selected and each point was divided into five soil layers (0 to 10 cm, 10 to 40 cm, 40 to 70 cm, 70 to 100 cm, and 100 to 150 cm, the deepest soil layer is 150 cm, as groundwater appeared approximately at 160 cm when the soil columns were dug out in the above selected OC and UC points). Each soil sample was dug with a soil auger between 4∶30 AM and 5∶30 AM (Xinjiang local time) in the middle of July, 2010. The nine soil samples for each soil layer from three points were mixed to makeup a composite soil samples. Meanwhile, on the periphery of selected plots, well water 3 m beneath ground level was collected from five sites, and subsequently mixed them to obtain a composite sample to replace groundwater. A river water sample was also mixed from three sites at least 1 km apart of the Aqikesu River. Each sample of river water was collected from 1 m beneath river surface. All the soil and water samples were immediately placed into glass bottles, sealed with Parafilm and stored in a mini refrigerator (4°C) and brought to the Xinjiang University Physiological Ecology Laboratory for further experimental analysis.

### Oxygen isotope measurement

Methods for extraction of soil water are consistent with Allison et al. [34]. Water isotope content (δ^18^O) was measured using a DELTA V Advantage Isotope Ratio Mass Spectrometer (Thermo, Waltham, MA, USA) at the Chinese Academy of Forestry Stable Isotope Laboratory. Each sample was measured three times continuously with the third result as the experimental oxygen isotope value. Precision values of continuous measurements for standard sample were as follows: D, <3‰ and ^18^O, <0.5‰. The isotopic abundance was expressed in delta notation (δ) in parts per thousand (‰) as
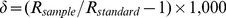
(1)Where Rsample and Rstandard are the molar ratios of heavy to light isotope of the sample and the international standard (Vienna standard mean ocean water for ^2^H/^1^H and ^18^O/^16^O) [1].

### Plots investigation

48 plots of UC were established having each plot size of 10×10 m (14 plots in June, 17 plots in August and October) in 5×5 km experimental plot from June to October, 2010. Consequently, 34 control plots with 10×10 m of OC (13 plots in June, 15 plots in August and 16 plots in October) were set at the same time. The species identification, richness, abundance, coverage, DBH, and crown area of each plant in each plot were investigated.

### Data analysis

To determine whether *P. euphratica* undergoes HR, a comparison analysis was used to show the difference in δ^18^O content for the five soil layers between the UC and OC groups. In addition, the paired-sample *t*-test was used to test the significant difference between above mentioned two groups.

Furthermore, based on the plots investigation data, the growth dominance index (Formulae 2 to 4) was used to show the growth condition, i.e. difference between UC and OC.
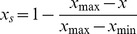
(2)

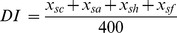
(3)

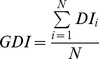
(4)Where *x*_s_, *x*_sc_, *x*_sa_, *x*_sh_ and *x*_sf_ are the standardized value, coverage, abundance, height and frequency for a given species in a given plot respectively. *x*_max_ and *x*_min_ are the maximum and minimum values for a given species in a given plot while *x* is the actual investigative value of each individual belonging to the same species in a given plot. *DI* is the dominance index in a given plot, and *GDI* is the growth dominance index of all plots. *N* is the count of plots, and *DI_i_* is the dominance index of a given species in *i* th plot.

Individual size has positive correlations with the amount of HR which can generally represent the water lift capacity of HR. It is well understood that the crown area of arid desert vegetation is significantly linked with adult size (tree height) and root biomass [35], [36]. Thus in this study, we used the individual crown area (CA, Formula 5) to represent water lifting capacity of *P. euphratica* HR. Maximum vegetation crown diameter (CD1) and its perpendicular diameter (CD2) were measured on each plant individual, which were used to calculate CA as follows (Formula 5).
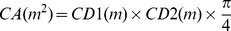
(5)


In order to explain the effects of HR on species richness and abundance in the arid desert community, an independent sample *t*-test was used between UC and OC to determine the diversity difference for shrubs and herbs. Finally, the logarithmic regression analysis was applied to explore the relationship of HR capacity (CA) to species richness (species number in 10×10 m) and abundance (count of individual plants in 10×10 m) for arid desert community.

All statistical tests were conducted using SPSS 11.5 while related figures were drawn using Origin 8.0. All statistical tests were considered significant at the *p*<0.05 level.

## Results

### Comparisons of δ^18^O values between the UC and OC

**Figure 1:**
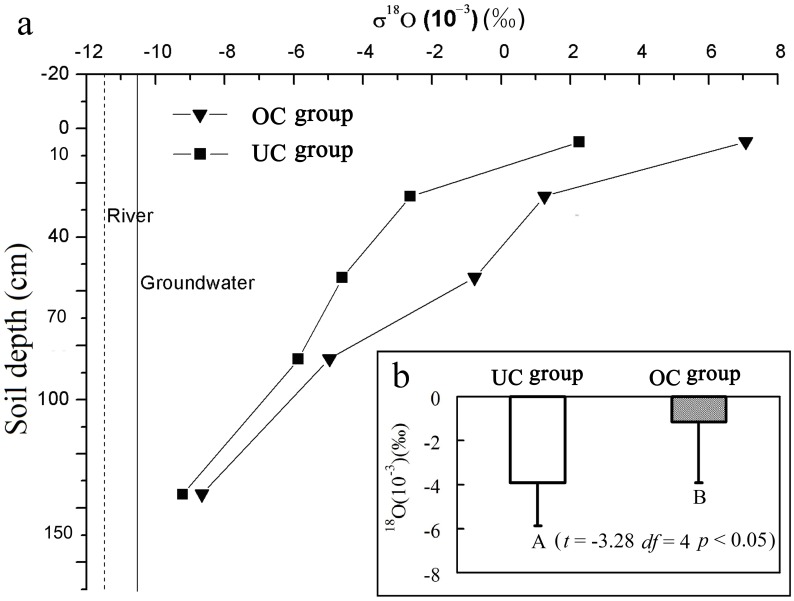
Difference in δ^18^O value with soil depth between UC and OC groups. UC is the community under the *P. euphratica* canopy, whereas the OC is the community outside the *P. euphratica* canopy. The soils were divided into five layers (0 to 10 cm, 10 to 40 cm, 40 to 70 cm, 70 to 100 cm, and 100 to 150 cm) based on *P. euphratica* roots and underground water level distributions. Each point in the Fig. 1a showing δ^18^O for each soil layer sample, which was measured for three times continuously with the third result as the experimental oxygen isotope value. Nine soil samples for each soil layer from three points were mixed to get one composite soil in each group. ^18^O contents of river (−11.55‰) and underground water (−10.59‰) are show through vertical dashed and solid lines respectively in Fig. 1a. Fig. 1b shows the mean comparison between UC and OC groups and the data in parenthesis showing the Paired-Sample *t*-test result. (Mean ± *SD*) for significance difference between two groups.

δ^18^O content of the OC were generally higher than that of the UC among five soil layers. In addition, the maximum difference between the two groups were observed in the surface soil layer (0 to 10 cm), and then decreased with soil depth (Fig. 1a). Therefore, a paired-sample *t*-test exhibited that the mean of five soil layer δ^18^O contents of OC was significantly higher than that of UC (*t* = −3.28, *df* = 4, *p*<0.05, Fig. 1b). The general comparison showed that δ^18^O content was the highest in soil, medium in groundwater and the lowest in river (Fig. 1a).

### Difference in species dominance index and biodiversity between the UC and OC

**Figure 2:**
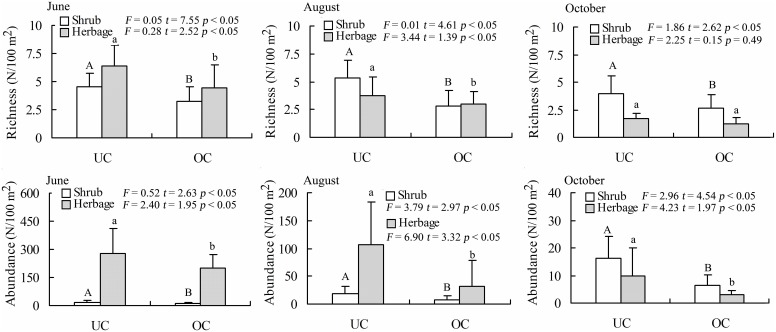
Difference in richness and abundance along plant growing season between UC and OC. UC is the community under the *P. euphratica* canopy, whereas the OC is the community outside the *P. euphratica* canopy. Blank and grid boxes indicate shrub and herbage, respectively. The Independent sample *t*-test is used to analyze the differences of richness and abundance between UC and OC. Different capital letters on each blank box indicate significant differences of shrub richness or abundance between UC and OC. Different lowercase letters on each grid box indicate significant differences of herbage richness or abundance between UC and OC. *p*<0.05. Numbers in figure are the results of Independent sample *t*-test. (Mean ± *SD*) for significance difference between UC and OC.

**Figure 3:**
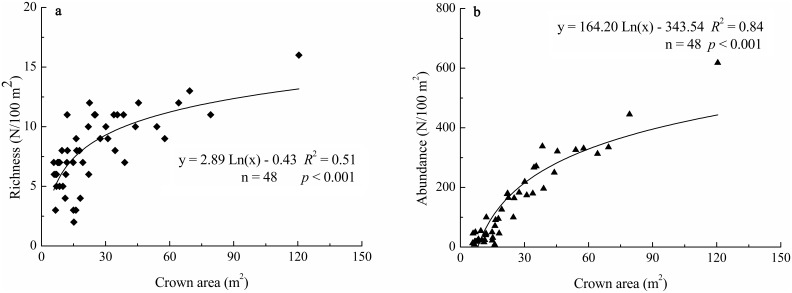
Logarithmic regression of *P. euphratica* crown areas against species abundance and richness. In regression line (Fig. 3a), each point indicating the richness of all species (no including *P. euphratica*) under the *P. euphratica* canopy. In regression line (Fig. 3b), each point indicating the abundance of all species (no including *P. euphratica*) under the *P. euphratica* canopy. The crown area of each sample is the sum of *P. euphratica* individuals.

Both the shrubs and herbs growth dominance index (*GDI*) of UC were higher than that of OC in June, August and October (Table 1). This finding indicates that the shrubs and herbs species grew better in UC than that in OC. Furthermore, compared with OC, the shrubs richness and abundance of the UC were significantly higher across three months (*p*<0.05) (Fig. 2). But for the herbs, this pattern of richness and abundance varies along the plant growing season. Between UC and OC, the species richness and abundance of herbs and shrubs were significantly similar in June and August, while non-significant differences were found in October (Fig. 2). Further, based on all UC plots investigated, the logarithmic regression analysis was used to analyze the relationship of *P. euphratica* individual HR capacities (individual crown area) with species richness and abundance. The results show that, species richness and abundance exhibited a significant exponent correlation with *P. euphratica* CA (*p*<0.05) (Fig. 3).

**Table 1:** Differences of species growth dominance index between the UC and the OC.

Life form	Species	Growth dominance index					
		June		August		October	
		UC	OC	UC	OC	UC	OC
Shrub	*Kalidium foliatum*	0.29	0.27	0.31	0.19	0.19	0.12
	*Reaumuria soogarica*	0.54	0.35	0.53	0.33	0.41	0.41
	*Alhagi sparsifolia*	0.67	0.50	0.29	0.22	0.34	0.25
	*Lycium ruthenicum*	0.23	0.21	0.35	0.32	0.26	0.09
	*Halostachys caspica*	–	0.06	0.26	0.33	0.16	0.21
	*Calligonum L*	0.15	0.08	0.05	–	0.05	0.05
	*Populus euphratica*	0.41	0.18	0.06	–	0.05	–
	*Nitraria schoberi*	0.20	0.13	0.12	–	–	0.05
	*Haloxylon persicum*	0.22	–	0.31	0.05	0.32	0.21
	*Salsola passerina*	0.05	–	0.20	0.03	0.15	0.05
	*Nitraria sibirica*	–	–	0.15	0.10	0.12	0.12
	*Tamarix ramosissina*	–	–	0.14	–	0.13	–
Herbage	*Karelinia caspicas*	0.42	0.25	0.14	0.11	0.05	0.05
	*Salsola soda*	0.67	0.51	0.54	0.45		
	*Salsola nitraria*	0.31	0.31	0.30	0.43	0.18	0.14
	*Poacynum henclersoni*	0.55	0.44	0.53	0.41	–	–
	*Spriphidium.*	0.34	0.11	0.09	–	0.05	–
	*Suaeda glauca*	–	–	0.22	0.10	0.17	0.05
	*Aeluropus littoralis*	0.07	0.06	0.05	–	0.05	–
	*Phragm ites australis*	–	–	0.17	0.30	0.15	0.05
	*Scorzonera austriaca*	0.11	0.18	0.10	–	–	–
	*Carpesium abrotanoides*	0.20	0.06	–	–	–	–
	*Afriplex patens*	0.08	0.06	–	–	–	–
	*Petrosimonia sibirica*	0.09	–	–	–	–	–
	*Malcolmia africana*	0.38	0.12	–	–	–	–

UC is the community under the *P. euphratica* canopy, whereas the OC is the community outside the *P. euphratica* canopy. For UC, *P. euphratica* is the only tree species (woody plant, height >6 m) while the others are shrub (woody plant, height <6 m) and herbage (herbaceous plants, height <1 m). For OC, all plants are shrubs and herbages. “–” in table indicating no values because of the ephemeral plants life history turnoff and randomly setting samples along June (early growth period of *P. euphratica*), August (middle growth period of *P. euphratica*) and October (defoliating period of *P. euphratica*). The figures in table showing Growth dominance indices of species belonged to UC and OC. All plots are established in 5×5 km area of Ebinur Lake Wetland Nature Reserve in Xinjiang Uygur Autonomous Region of China.

## Discussion

### Experimental validation of *P. euphratica* HR

Oxygen isotope fractionates during water transport between the different soil layers because of physical and chemical adsorption and conduction processes, which changes the ^18^O/^16^O values in different soil layers. However, the fractionation of oxygen isotopes does not occur during water transport through the xylem vessels [1], [26]–[28]. So the water originating from the deeper soils or groundwater in the shallow soils is an indicator for HR occurrence [1]–[3], [11], [37]. In the present study, the soil samples of UC and OC groups were collected at between 4∶30 AM and 5∶30 AM (local time), during which the fractionation not occur due to soil evaporation. Therefore, if *P. euphratica* HR does not occur, as the environmental conditions were same between UC and OC groups, the δ^18^O content of the corresponding soil layers did not differ significantly between UC and OC groups. However, in this study, all δ^18^O content of UC were lower than those of OC group among five soil layers (Fig. 1a), and the mean of UC was also significantly lower than OC group (Fig. 1b). This overall pattern exhibited that the water of all UC soil layers partially originated from deeper soil or groundwater through plants vessel transport. In other words, the deep-rooted *P. euphratica* exhibited HR, which transported and lifted water from deeper to shallow soil layers, proof for the experimental support to Ryel model based studies [22].

It is well understood that changes in soil evaporation depends on vegetation coverage, for example, the higher vegetation coverage will has more shade on the soil surface and hence evaporation will decrease, and vice versa [38], [39]. Furthermore, Allison [40] and Kim [41] confirmed that δ^18^O content of soil samples in UC is lower as compared to OC, because high vegetation coverage decreased the evaporation due to shading. Specifically, higher evaporative demand in OC could easily drive a greater upward movement of water from the groundwater or the deeper soils to surface soils, which then enriched the δ^18^O content due to evaporative fractionation. However, pan evaporation was not significantly different between UC and OC (UC_soil evaporation_ = 0.91±0.52 cm·m^−2^·d^−1^, OC_soil evaporation_ = 0.93±0.55 cm·m^−2^·d^−1^, *t* = −1.02, *df* = 6, *p* = 0.34, which was tested through paired-sample *t*-test, and its supportive data was measured by 255 Series Evaporation Stations (EP255, Novalynx Inc, OR, USA) for three paired groups in UC and OC), because the sparse vegetation coverage of *P. euphratica* community (approximate 4%) has little shading influence on the evaporative demand of UC and OC in Ebinur desert [22]. In addition, this desert is a part of tuyere zone having high wind, and thus other evaporative environmental factors (*e.g.*, air temperature and moisture) homogenously influence soil evaporation, subsequently caused relatively no difference of soil evaporation between UC and OC plots. Hence, soil evaporation not likely differed δ^18^O content between OC and UC in the Ebinur desert and further proved that *P. euphratica* HR occurs.

*P. euphratica* HR and water transport direction along soil profile can also be judged by moisture differences among soil layers. In this study, Thetaprobe ML2 soil moisture sensors (Delta-T Devices, Cambridge, UK) were installed respectively in five soil layers of UC and OC to measure the variation in soil volumetric water content (SVWC) along soil depth. The results showed that, (1) SVWCs of 0 to 10 cm, 10 to 40 cm, 70 to 100 cm and 100 to 150 cm layers in UC were significantly higher than those in OC, except in 40 to 70 cm soil layer; (2) SVWC increases with soil depth but decreases with δ^18^O content in OC, while no significant trend was found in UC; and (3) SVWC of 100 to 150 cm soil layer was a little higher in OC than in OC (Table 2). These results suggested that groundwater supplies soil water, and the water’s table in both UC and OC were similar in Ebinur desert. SVWC increased with soil depth due to the extraction of soil evaporation in OC. But HR can lift water from groundwater and deep soils to shallow soils and then decrease the effect of evaporation on water extracting in UC. Previous studies showed that the location and the amount of HR releasing water were significantly depended on plant fine roots distribution [11], [22], [23]. Also *P. euphratica’*s fine roots was mainly growing within 0 to 70 cm soil depth in arid area [22], [29]. Thus, HR may cause no change in SVWC across soil depth in UC and SVWC of 40 to 70 cm layer in UC less than that in OC.

**Table 2:** Variation in soil volumetric water content across soil depth and δ^18^O content in UC and OC.

Soil layers (cm)	δ^18^O (‰)		Soil volumetric water contents (Mean ± *SD*) (cm^3^/cm^3^)				
	UC	OC	UC	OC	*F*	*t*	*P*
0–10	2.27	7.10	8.41±0.38A	3.86±1.67B	45.60	21.79	<0.001
10–40	−2.62	1.25	29.72±0.41A	18.86±0.26B	1.95	181.58	<0.001
40–70	−4.60	−0.77	12.63±0.45A	20.26±0.35B	16.03	−109.46	<0.001
70–100	−5.87	−4.98	23.07±0.40A	21.40±0.07B	2.08	34.07	<0.001
100–150	−9.21	−8.67	27.98±0.03A	27.55±0.02B	66.78	7.49	<0.05

UC is the community under the *P. euphratica* canopy, whereas the OC is the community outside the *P. euphratica* canopy. UC and OC have three measuring plots, respectively. The Independent sample *t*-test is used to analyze the differences of soil volumetric water content between UC and OC. Different capital letters in each row indicate significant differences of soil volumetric water content between UC and OC. The measurement period of soil volumetric water content lasts from 5th to 8th August, 2010. During the time between 2:30 am to 6:30 am, 11∶30 am to 5∶00 pm at each experimental day, the soil volumetric water content recorded manually at intervals of 2 hours, while the time between 6:30 am to 9:30 am, 9:30 pm to 12:30 am, the soil volumetric water content recorded manually once an hour.

The greatest δ^18^O content existed in soil while the intermediate in groundwater and the lowest in river (Fig. 1a). This pattern indicates that river is the initial source of water for arid desert area. Our results were supported by Zhao et al. [42] reported that river largely originated from glacial melting and has the lowest δ^18^O content in arid desert. Based on oxygen isotope fractionation theory [26]–[28], [40], initial water resource has lowest δ^18^O content and then increased with transmittal distance and pathways. In this study, the river is the main source for groundwater and then supply to soil water through water transmittal ways. i.e. underground or surface runoff, evaporation and HR. All of those can led to oxygen isotope fractionation and then resulted in soil having the greatest value of δ^18^O content.

### Effects of *P. euphratica* HR on species growth condition

The importance value index [importance value index = (relative abundance + relative frequency + relative coverage + relative height)/400] and its deduced dominance index (dominance index = 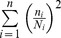, *N* is the count of plots and *N_i_* is the importance value of a certain species in *i* th plot) were traditional ecological methods in generally using to evaluate species growth conditions within a specific community type [43], [44]. Specifically, the importance value is commonly assumed as 1, and then based on individual relative statistical values, such as individual numbers, relative abundance, relative coverage, relative frequency, and relative height. 1 was divided into different components values that show the different species growth conditions within a given community. Nevertheless, many studies also needed to compare the difference of same species growth conditions among environmental sites, such as the same species between UC and OC. According to this situation, considering *P. euphratica* can account for the largest partition of the importance value in UC, if we used the traditional methods above to evaluate the difference of species growth conditions between UC and OC, a lower importance value could be found in UC than in OC among other species, even these species have the same abundance, coverage, frequency and height between two environmental types.

In this study, the growth dominance index was structured by the actual values of coverage, abundance, height, and frequency of a given species in a given plot to avoid the components influence of importance value, and to compare the difference of species growth conditions between the UC and OC (Formulae 2 to 4). The results showed that UC species grow better than OC along plant growing seasons (Table 1). Additionally SVWC increased with soil depth in OC while no change in UC (Table 2). These suggest that *P. euphratica* HR increased significantly water content of shallow soils and benefited the growth condition and survival of shallow-rooted plants [16], [17], [24], [37], [45]–[50].

### Relationship between *P. euphratica* HR and arid desert diversity assembly

Water is the main limitation for arid desert plant communities [1], [51]. Thus, species coexistence pattern in this community directly depends on spatial availability and distribution of water [32], [33]. In this study, the species richness and abundance in the UC were significantly higher than those in the OC (*p*<0.05), i.e., UC has higher species diversity (Fig. 2).

Furthermore, based on the hypothesis of this study that a tree with a larger crown area has higher root biomass and transports more water from the deep to shallow soils, the logarithmic regression was used to analyze the relationship between the *P. euphratica* HR capacity (CA) and the species richness and abundance in the UC. The results showed that the *P. euphratica* CA was significantly logarithmically correlated with richness (*R^2^* = 0.51) and abundance (*R^2^* = 0.84) (*p*<0.001) (Fig. 3). These results indicated that species diversity was influenced significantly by HR. Similarly, this conclusion was also reflected in other studies. For example, herbaceous plants under tree canopies have higher abundance and richness [46], [52], and the presence of trees promoted the survival of shrubs and seedlings grown under tree canopies [37], [45], [48]–[50], [53].

Shallow-rooted herbs and shrubs that absorb water from *P. euphratica* HR provided more organic matter and mineral elements to the soil of the UC than to those of the OC because of the “fertile island” effect. It implies that more individuals of the UC are intercepted and sequester larger amounts of organic matter and minerals from surface winds because of increased total crown area [54]–[59]. Meanwhile, the more numerous individuals also produced more litter into the UC soils than OC soils. Thus, these processes contributed to the nutrient absorption and growth of *P. euphratica*, which further increased its HR capacity. In turn, these processes also increased the shading in understory plants, which can decrease the transpiration of herbs and shrubs, and further improve its survival. Therefore, there appears to be a species coexistence pattern of water sharing and resource complementation between the deep-rooted *P. euphratica* and other shallow-rooted species, as well as positive feedback between *P. euphratica* HR and biodiversity maintenance in arid deserts community.
